# Associations between Dopamine D4 Receptor Gene Variation with Both Infidelity and Sexual Promiscuity

**DOI:** 10.1371/journal.pone.0014162

**Published:** 2010-11-30

**Authors:** Justin R. Garcia, James MacKillop, Edward L. Aller, Ann M. Merriwether, David Sloan Wilson, J. Koji Lum

**Affiliations:** 1 Laboratory of Evolutionary Anthropology and Health, Binghamton University, Binghamton, New York, United States of America; 2 Department of Biological Sciences, Binghamton University, Binghamton, New York, United States of America; 3 Department of Anthropology, Binghamton University, Binghamton, New York, United States of America; 4 Institute for Evolutionary Studies, Binghamton University, Binghamton, New York, United States of America; 5 Department of Psychology, University of Georgia, Athens, Georgia, United States of America; 6 Center for Alcohol and Addiction Studies, Brown University, Providence, Rhode Island, United States of America; 7 Department of Psychology, Binghamton University, Binghamton, New York, United States of America; 8 Department of Human Development, Binghamton University, Binghamton, New York, United States of America; Kyushu University, Japan

## Abstract

**Background:**

Human sexual behavior is highly variable both within and between populations. While sex-related characteristics and sexual behavior are central to evolutionary theory (sexual selection), little is known about the genetic bases of individual variation in sexual behavior. The variable number tandem repeats (VNTR) polymorphism in exon III of the human dopamine D4 receptor gene (DRD4) has been correlated with an array of behavioral phenotypes and may be predicatively responsible for variation in motivating some sexual behaviors, particularly promiscuity and infidelity.

**Methodology/Principal Findings:**

We administered an anonymous survey on personal history of sexual behavior and intimate relationships to 181 young adults. We also collected buccal wash samples and genotyped the DRD4 VNTR. Here we show that individuals with at least one 7-repeat allele (7R+) report a greater categorical rate of promiscuous sexual behavior (i.e., having ever had a “one-night stand”) and report a more than 50% increase in instances of sexual infidelity.

**Conclusions/Significance:**

DRD4 VNTR genotype varies considerably within and among populations and has been subject to relatively recent, local selective pressures. Individual differences in sexual behavior are likely partially mediated by individual genetic variation in genes coding for motivation and reward in the brain. Conceptualizing these findings in terms of r/K selection theory suggests a mechanism for selective pressure for and against the 7R+ genotype that may explain the considerable global allelic variation for this polymorphism.

## Introduction

Human sexual behavior varies dramatically across human populations, including patterns of promiscuity and sexual infidelity [1]–[3]. While mating and pair-bonding are human universals [1], [4], individuals also engage in uncommitted short-term sexual activities at varying rates [1], [5]. Yet, the proximate mechanisms governing the individual differences in proclivities for uncommitted sexual behavior are unclear. In the current study we explore the role of individual genetic variation in influencing motivations for uncommitted sexual activity. We conceptualize sexual promiscuity as uncommitted sexual intercourse with non-monogamous partners (i.e., “one-night stands”) while sexual infidelity includes any physical sexual activity with an individual other than one's current self-identified committed relationship partner (i.e., “cheating”). We conceptually differentiate sexual (genetic) monogamy from social (pair-bonded) monogamy, and as such recognize that infidelity is a particular instantiation of uncommitted sex where an individual is actually traditionally committed, however, not to a partner they are engaging in sexual activity with.

### Ultimate Mechanisms

Beyond humans, other socially “monogamous” animals also engage in sexual activity with conspecifics they are not pair-bonded to. A vast majority of birds form pair-bonds for the production and rearing of offspring, yet in more than 70% of species reviewed, at least some of the genetic offspring result from extra-pair copulations [6]–[7]. One extensive field study of the purportedly socially monogamous swift fox (*Vulpes velox*) reports that over 50% of females' offspring were genetically sired by males other than the partner to which she was pair-bonded [8]. And among primates, both males and females of the gibbon family (*Hylobatidae*) engage in extra-pair copulations despite strong pair-bonds [9]. Evolutionarily, such behaviors are presumably for the purposes of increased offspring diversity, increased offspring genetic quality, and/or extra fertilizations.

In humans, measures for global and national rates of promiscuity and infidelity are more varied. Worldwide median rates of non-paternity, or the rate of men raising children under the pretense of biological parentage, have been suggested to near 9% [10], or 10% more popularly [11]. However, this rate is mitigated by a male's degree of paternity certainty, with men who display high paternity confidence having a lower actual median non-paternity rate of 1.7% [11]. Further, an implicit condition of mate poaching, the active seduction of an individual away from a partner with whom they are romantically involved [3], generally includes infidelity on the part of the one being poached from their partner. Although mate poaching occurs as a “cultural universal” across no less than fifty-three previously studied nations [3], the frequency of mate poaching and all forms of uncommitted sexual behaviors vary widely among populations, reflecting differences in both local social constraints and individual behaviors [1]–[3], [5].

Individuals engaging in promiscuous sexual behaviors also face substantial risks, including diseases, unwanted pregnancies, and adverse social consequences. In addition, individuals engaging in infidelity also risk potentially losing or angering their pair-bonded partner. Historically and cross-culturally legal codes have responded to this evolutionary threat with biases accommodating entitlement to crimes of passion following evidence of adultery [12]. Despite these risks, sexual behavior in general and infidelity in particular offers enormous proximate and ultimate benefits in terms of immediate psychophysical reward and contributions to increased total number of offspring and subsequent increased diversity amongst those offspring. Given the potential disadvantages and advantages for social status and biological fitness, sex-related characteristics and sexual behavior itself are subject to substantial conflicting selective pressures [1]–[2], [5].

The substantial heritabilities between female monozygotic twins of 41% for number of lifetime sexual partners and 38% for infidelity demonstrates the contribution of genetics to these sexual behaviors [13]. Among monozygotic and dyzygotic twins of both gender, risky sexual behavior was correlated with personality traits and suggested heritability of 33% [14]. Further, high male reproductive variance enabled by promiscuity and infidelity of *both* sexes, irrespective of social monogamy, may be essential for the rapid fixation of beneficial genes within a population.

### Proximate Mechanisms

Functionally, the brain's dopamine reward system supports motivation and libido to influence sexual and pair-bonding behaviors [15]–[17]. Dopaminergic function is enhanced by oxytocin and arginine vasopressin acting on the hypothalamus to regulate attachment [15], [17]–[20]. More specifically, it is oxytocin and vasopressin which most influence monogamy and pair-bonding [16], [18]–[20]. This is supported by recent genetic evidence of the vasopressin receptor 1a gene (AVPR1A) being associated with pair-bonding and marital satisfaction [21]. However, in a sample of female twins this same gene (AVPR1A) was not associated with infidelity or number of sexual partners [13]. Pair-bonded relationship satisfaction is not *necessarily* linked to promiscuity and infidelity, as the underlying motivations may be quiet different both psychosocially and neurobiologically. In fact, over two-thirds of adulterous men report that they would never have predicted their own infidelity [22]. Rather than envisioning relationship stability and infidelity as opposite ends of the same continuum, the underlying biological motivations for such behaviors may well be associated with distinct neurobiological pathways and thus more accurately thought of as orthogonal.

Additional studies from behavioral neurogenetics suggest that genes modulating dopamine neurotransmission mediate a variety of behavioral phenotypes associated with sensation-seeking [23]–[27]. One specific candidate is the dopamine D4 receptor (DRD4) gene, with a 48bp variable number tandem repeat (VNTR) polymorphism in exon III of chromosome 11. This polymorphic region typically includes 2 to 11 repeats. Individuals with at least one allele containing 7 or more repeats (7R+) show both reduced binding affinities and receptor densities for dopamine neurotransmission in the ascending corticomesolimbic reward pathway that extends from the ventral tegmental area to the nucleus accumbens, prefrontal cortex, and other cortical regions [28]. Individuals with these long alleles are predisposed to sensation-seeking behaviors including both migration and novelty-seeking, resulting in differential survival and reproduction [23], [25]–[26], [29]. The dopaminergic reward pathway influences physiological arousal, pleasure, and intrinsic reward [30]. Humans that possess at least one allele 7-repeats or longer (7R+) display behavioral phenotypes associated with attention deficit hyperactivity disorder (ADHD) [31], alcoholism [32]–[33], financial risk-taking [34], disinhibition and impulsivity [24], and sexual behavior [35], [36]. The latter includes associations between DRD4 genotype and sexual desire, arousal, and function [36], as well as likelihood of initiating sexual activity among young adults [35]. DRD4 7R+ has also been reported as unrelated to number of previous sexual partners [37], however the high reward-seeking variant of the dopamine transporter gene DAT1 was associated with increased number of sexual partners [38], although only in males. There is also support for an association between DRD4 7R+ and multiple/interracial ancestries [39]. DRD4 VNTR genotype frequencies vary widely across the globe, reflecting the adaptive utility of both motivations for dispersal [23] and in some cases culturally constrained behavioral phenotypes [26]. Taken together this suggests that the relationships between sexual behavior, human evolution, and dopamine modulating genes are fairly nuanced. Given the important role of dopamine in sexual behavior [17], it is predicted that variation in DRD4 will be associated with uncommitted sexual behavior in men and women.

## Methods

### Participants and Procedures

All aspects of this research were conducted in accordance with guidelines for the use of humans as research participants, and was approved by the University's AAHRPP accredited Human Subjects Research Review Committee. All participants provided written and verbal informed consent before initiating the study. Participants were 181 young adults (118 females, 63 males) recruited from a midsized state university in the northeastern United States. Average age of participants was 20.11 years (SD  = 1.62). Participants were primarily of European ancestry (61%), with 19% of Asian ancestry, 9% Hispanic, 1% African-American, 4% multi-racial, and 6% “Other.” Participants were recruited through the Psychology Department Human Subjects Research Pool and attended group assessment sessions seated every other seat and row with alternating order/color packets to ensure both actual and self-perceived privacy while they completed a confidential survey relating to sexual behavior and provided a DNA sample.

### Phenotypic Assessment

The self-report survey included a comprehensive measure of demographics and questions on past sexual behavior, sexual expectations, and preferences, within which the primary dependent variables of interest were assessed. Participants were also assessed for smoking habits using the Fagerström Test of Nicotine Dependence (FTND) [40] and delayed reward discounting (DRD) using the Monetary Choice Questionnaire (MCQ) [41]. The MCQ is a self-report measure of DRD, which is an index of impulsivity, reflecting preferences for smaller immediate rewards compared to larger delayed rewards. In this case, participants made hypothetical choices for smaller amounts of money immediately versus larger amounts after a period of days (e.g., would you prefer $25 today, or $60 in 14 days); the MCQ provides indices of overall discounting as well as discounting for small, medium, and large rewards. Both the FTND and MCQ have been psychometrically validated.

### Genotyping

For DNA analysis, a non-invasive oral buccal wash sample was obtained using 10 ml of Scope™ mouthwash [42]. Sufficient DNA for DRD4 PCR amplification was extracted from 96% (173/181) of the buccal cell samples. DNA was extracted using an abbreviated silica extraction protocol.

The human DRD4 gene on chromosome 11 contains a 48bp variable number tandem repeat (VNTR) polymorphism in exon III. Previous studies have highlighted problems associated with consistent genotyping of the DRD4 VNTR region [43], suggesting multiple PCR and electrophoresis runs for each sample to control for allelic dropout. Thus, the PCR reaction was modified to reflect the high GC content (see below) and all samples that were initially scored as homozygotes were reanalyzed two additional times with different starting template concentrations to unambiguously confirm genotypes. The PCR reaction consisted of 1x Q-Solution (Qiagen), 1x Buffer (Qiagen), 1 µM Primer 1 (5′ GCGACTACGTGGTCTACTCG 3′), 1 µM Primer 2 (5′ AGGACCCTCATGGCCTTG 3′), 200 µM dATP, 200 µM dTTP, 200 µM dCTP, 100 µM dITP, 100 µM dGTP, 0.3 units HotStar Taq (Qiagen), and 1 µl of DNA template, in a total volume of 10 µl. The PCR profile began with 15 minutes at 95°C for enzyme activation and denaturing of template DNA followed by 40 cycles consisting of 1 minute denaturation at 94°C, 1 minute annealing at 55°C, 1.5 minute extension at 72°C, and finished with a 10 minute extension at 72°C. Amplicons were electrophoresed through 1.4–2.0% agarose gels containing ethidium bromide and genotypes were determined by comparison with a 100 bp ladder.

### Data Analysis

The primary dependent variables were history of sexual intercourse, infidelity (“cheated on” a committed partner), and promiscuity (“one-night stands”). In each case, categorical (i.e., Yes/No) and continuous (i.e., number) assessments were made. Nonparametric statistics and generalized linear model (GLM) analyses yielded largely parallel results, but for brevity only GLM results are shown when appropriate. Exact *p* values are reported for consideration by future studies. For continuous variables, medians with interquartile ranges are provided as measures of central tendency.

To facilitate truthful responding and per the Human Subjects Research Review Committee, participants were permitted not to answer individual items if they were uncomfortable given the personal nature of the assessment. This resulted in small portions of absent data: history of sexual intercourse, 8%; one night stands, 10%; infidelity, 4%. Genotypes were grouped as 7R+ (at least one allele 7-repeats or longer) or 7R- (both alleles less than 7-repeats); the 7R+ genotype was present in 24% of the sample. The analyses comprised three approaches, the primary approach and two strategies for ruling out alternative explanations. The primary analyses examined the relationship between the phenotypes of interest and DRD4 VNTR status. Second, possible contributions of population stratification were examined by re-running the analyses within the largest homogeneous group in terms of racial ancestry. Finally, the relationships among DRD4 VNTR genotype, sex, age, sexual behavior, smoking behavior, and impulsivity (DRD) were examined to determine the specificity of the associations in the primary analyses.

## Results

### Sexual Behavior and DRD4 VNTR Genotype

**Figure 1:**
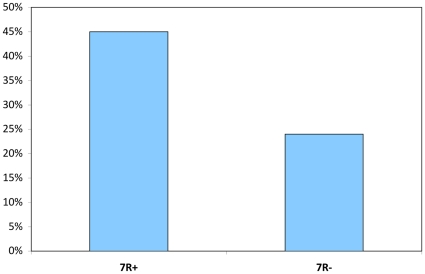
Percent who report promiscuous sexual experiences, by DRD4 genotype group.

**Figure 2:**
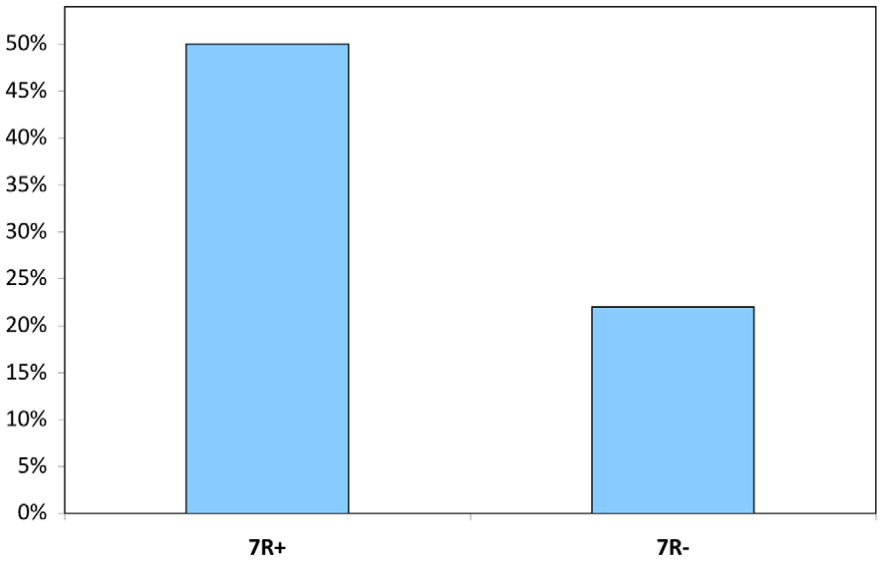
Percent who report extra-relationship sexual experiences, by DRD4 genotype group.

**Figure 3:**
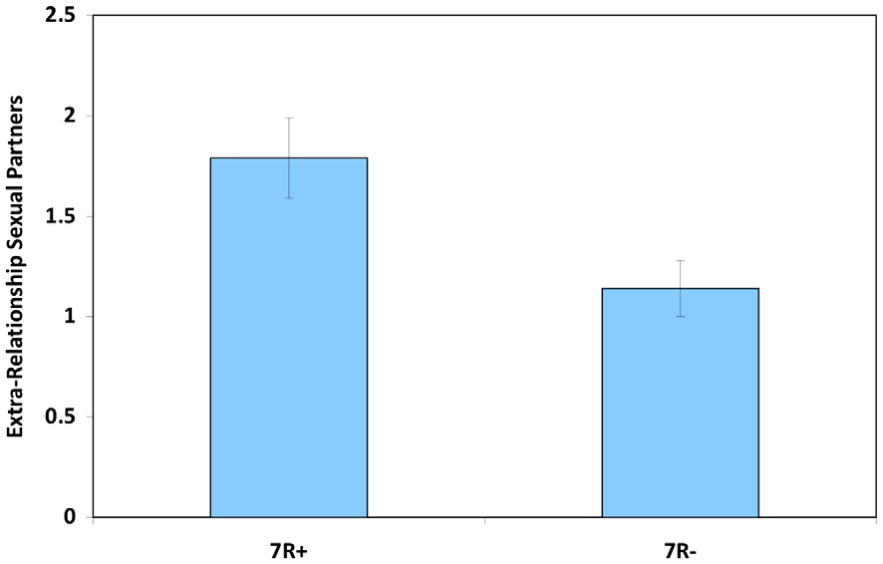
Number of extra-relationship sexual partners, by DRD4 genotype group.

Seventy seven percent of the sample reported a history of sexual intercourse, and no differences were evident between the genotypic groups (χ^2^ [df  = 1]  = 0.32, *p* = 0.57). Among those who reported previous sexual experience, there was no genotypic difference in terms of the total number of sexual partners (*F* [1, 125]  = 1.59, *p* = 0.21). In contrast, categorical rates of promiscuous sex differed significantly between 7R+ and 7R- individuals (χ^2^ [df  = 1]  = 5.58, *p* = .018). This difference reflected an almost 2-fold greater promiscuity rate in 7R+ (45%) compared to 7R- (24%) individuals (see Figure 1). However, no significant differences were evident in the total number of reported instances of promiscuity between the two genotypic groups among those who reported promiscuous sex (*F* [1], [33]  = .30, *p*  = .59). A similar pattern was evident for sexual fidelity, where 50% of 7R+ individuals reported being unfaithful compared to only 22% of 7R- individuals (See Figure 2), although this difference fell short of statistical significance (χ^2^ [df  = 1]  = 2.15, *p* = .14). However, among individuals who reported sexual infidelity, 7R+ individuals reported significantly more extra-pair copulation partners (mean  = 1.79, SEM  = .20) compared to 7R- (mean  = 1.14, SEM  = .14) individuals (*F* [1], [40]  = 7.08, *p* = .011). Taken together, 7R+ were almost twice as likely to have engaged in promiscuous sex, and, when they were unfaithful, 7R+ individuals reported more than 50% more extra-pair copulation partners than 7R- individuals (See Figure 3).

### Sample Racial Ancestry Characteristics and Population Stratification

To examine possible population stratification effects (a “chopsticks gene”) [44], the preceding analyses were re-run in the largest racially homogenous ancestry (i.e., European, 61%), and all significant and nonsignificant findings were replicated. Participants were categorized racially according to their self-report of the racial/ethnic ancestry of all four of their grandparents. Group membership was assigned based on all four grandparents being of the same ancestry or being of multiple ancestries.

Within individuals of European ancestry, 7R+ individuals were more likely to report having engaged in promiscuous sexual behavior (χ^2^ [df  = 1]  = 4.24, *p* = .04), but, among those who reported at least one promiscuous episode, no difference in the number of instances (*F* [1], [18]  = .21, *p* = .65). Also consistent with the primary findings, 7R+ individuals were no more likely to have been unfaithful (χ^2^ [df  = 1]  = 1.60, *p* = .21), but, among those who reported infidelity, 7R+ reported a larger number of extra-pair copulation partners (*F* [1], [26]  = 5.83, *p* = .023). This is the same pattern as the total sample, suggesting systematic differences by racial/ethnic ancestry did not contribute to these findings.

### Roles of Sex (Gender), Smoking Status, and Impulsivity

No sex differences were evident in terms of genotype frequency (female 7R+ frequency  = 23%, male 7R+ frequency  = 26%), suggesting it is unlikely that the findings are a function of systematic sex differences (χ^2^ (df  = 1)  = .20, *p* = .66). Similarly, genotype frequency did not systematically vary by age (*F* [1, 167]  = 1.64, *p*  = .20) and age was not associated with continuous measures of promiscuity (Pearson *r* = .02, *p* = .90) or infidelity (Pearson *r* = .13, *p* = .40). These analyses suggest that participant age did not play a role in the primary findings. Genotype status was not associated with dichotomous smoking status (χ^2^ [df  = 1]  = 1.12, *p* = .29). Interestingly, however, among cigarette smokers, 7R+ individuals had higher levels of nicotine dependence (mean  = 3.22, SEM  = .50) compared to 7R- (mean  = 1.47, SEM  = .34; *F* [1], [26]  = 8.32, *p* = .008). However, FTND was associated with neither instances of promiscuity (Pearson *r* = .02, *p* = .95), nor instances of infidelity (Pearson *r* = −.02, *p* = .97), suggesting independent relationships. Taken together, this suggests that the findings were not attributable to smoking status or nicotine dependence. Finally, DRD4 VNTR genotype was not associated with delayed reward discounting in general (F [1, 158]  = .60, *p* = .44), or for large (F [1, 158]  = .83, *p* = .37), medium (F [1, 158]  = .28, *p* = .60), or small (F [1, 158]  = .51, *p* = .48) rewards, further suggesting that the primary findings were not attributable to a more general preference for smaller immediate rewards over larger delayed rewards.

## Discussion

These results are the first evidence (to our knowledge) of a significant association between a specific genetic locus and both promiscuous sexual behavior and infidelity. These findings show that genetic variation in the brain′s dopaminergic reward pathway appears to be an influential factor in individual differences in motivation to engage in sexual behavior of a risky and uncommitted nature. Further, this potentially suggests an evolutionary mechanisms contributing to the substantial global allelic variation of the DRD4 VNTR genotype. Individuals genotyped as 7R+ were significantly more likely to reported having ever engaged in promiscuous sex (i.e., a one-night stand). Of those reporting infidelity, 7R+ individuals were cheating on romantic partners more often, which under certain circumstances could result in higher genetic fitness via greater offspring diversity as well as increased total fecundity. This suggests that in local environments where monogamy and sexual fidelity are advantageous, the 7R- genotype would be subject to positive selective pressure. In contrast, in environments where monogamy and fidelity are disadvantageous, the 7R+ genotype would be subject to positive selective pressure. This may be additionally elucidated from the perspective of r/K selection [45]. In r-selected environments (i.e., unpredictable and unstable environments, where the ability to mate more and produce more offspring is favored), 7R+ genotype would be expected to rise in frequency. That is, in environments where “cad” behavior is adaptive, selective pressure for 7R+ would be positive; but in environments where “dad” behavior is adaptive, selective pressure for 7R+ would be negative. This is consistent with the dramatic differences in DRD4 VNTR allele frequencies and behavioral patterns found globally such as in the generally polygamous and agonistic Yanomamö Indians of South America (high 7R+ frequencies) and the generally egalitarian !Kung of the Kalahari (low 7R+ frequencies) [26]. Evidence that DRD4 VNTR status is related to social and sexual behavioral strategies provides a plausible mechanism for varying selective pressure and observed racial, ethnic, and regional differences in allele frequencies.

DRD4 VNTR variation has been associated with a wide array of behavioral tendencies and psychiatric conditions. Among the most consistent are the association between 7R+ and ADHD [31] and that 7R+ individuals exhibit augmented anticipatory desire response to stimuli signaling dopaminergic incentives, such as food, alcohol, tobacco, gambling, and opiates [27], [32]–[34]. Although it is as yet speculative, these associations suggest that 7R+ individuals may allocate greater attention to appetitive rewards, contributing to the behavioral differences in promiscuity and infidelity observed here.

Important as this finding may be, it is also important to sound several notes of caution. First, a consistent challenge in genetic association studies are that of third variable confounds, or unmeasured variables that are causally responsible for the observed finding but are associated with the measured variables thus generating a spurious association. In this case, such third variables could be additional genetic loci in partial or complete linkage with the DRD4 VNTR variation, or alternative unmeasured phenotypes that are likewise associated with the dependent variables. We attempted to rule out some such alternative possibilities in this study. Population stratification is one example, but did not play a role insofar as the findings were robust within the largest group of self-reported racial ancestry. We also examined gender, age, impulsivity, and substance use in the form of nicotine dependence, with no evidence that the results were better accounted for by those factors. However, it is nonetheless possible that other unmeasured third variables could have played a role. For example, it is conceivable that 7R+ individuals may be more likely to be forthright about their sexual behavior, inflating the associated rates compared to 7R- individuals. Alternatively, the genotype groups within this study could have systematically varied in dimensions of attractiveness (actual or perceived) or other mating-relevant aspects that may have influenced the results. As such, and as the first report of this association, it is important to recognize that alternative explanations remain possible and these findings should not be considered definitive at this point.

Presuming this association is robust across samples in future studies, a second reason for caution is that the behavioral outcomes examined are *probabilistic* and by no means deterministic. That is, our findings suggest higher rates and instances of the behaviors assessed, but not that all individuals who are 7R+ or 7R- will necessarily exhibit the behavioral outcomes associated with each genotype. For example, as is noted, about a quarter of the 7R- individuals reported promiscuous sex, which was a significantly lower rate than the 7R+ individuals but by no means trivial. Given general reasons to be cautious in behavioral genetic research and the inherently probabilistic relationship observed, we emphasize that it would be prudent to avoid premature and facile characterizations of the DRD4 VNTR polymorphism as “the promiscuity gene” or “the cheating gene.” Looking forward, it will be essential to replicate these findings and confirm genotypic and phenotypic specificity. If it is robust, an important priority will be on “connecting the dots” between genotype and variation in human sexual behavior by identifying the proximal neurobiological and behavioral mechanisms that underlie the genetic influences.

It should also be noted that recent historical (both biologically and socially influenced) shifts in sexuality discourse and rates of sexually permissive activity result in frequent uncommitted sexual behaviors among young adults today [46]. While sexual reproduction remains the currency of evolution, cultural regulation of sexual behaviors constrain the evolutionary best interests of individuals within a population. We suspect that the associations we observed between dopaminergic sensation-seeking and sexual behavior may be independent of other evolved mechanisms that promote pair-bond stability and romantic attachments. That is, the motivation to engage in extra-relationship sexual experiences (infidelity) or promiscuous sexual activities (one-night stands) can remain disconnected from any motivation for attachment and commitment even in the presence of strong existing pair-bonds. Characterizing the neurogenetic bases of diverse forms of sexual behavior will be essential in future studies that wish to elucidate the evolutionary and biocultural determinants of human behavior and sexuality.
